# MINNESOTA’S 2021 LGBTQ AGING NEEDS ASSESSMENT

**DOI:** 10.1093/geroni/igac059.2158

**Published:** 2022-12-20

**Authors:** Rajean Moone

**Affiliations:** University of Minnesota, St Paul, Minnesota, United States

## Abstract

Every decade Minnesota embarked on a needs assessment of lesbian, gay, bisexual, transgender and queer (LGBTQ) older adults which has provided important insights into aging within these communities. In 2021, a research team replicated these past monumental studies into this Minnesota 2021 LGBTQ Aging Needs Assessment. 485 individuals responded to the survey, either online or by paper. Of those, 354 met the inclusion criteria and are included in the results. Like the 2012 study, LGBTQ older adults who participated in the study were more likely to be a caregiver than compared to the general population. At the same time they were less likely to have a caregiver and less likely to have children. Half of the participants experienced some form of discrimination and the vast majority more knew someone who experienced discrimination due to their sexual orientation or gender identity. Positively, study participants were more likely to have completed a health care directive and more likely to volunteer than the general population. Possibly one of the most striking changes over the last two decades is that 85% of respondents were confident they would receive sensitive services. In 2012 only 18% and 2002 only 9% expressed the same confidence. In congruence with the past studies, the majority of respondents continue to desire LGBTQ welcoming senior services rather than LGBTQ segregated services.

